# Web-Based Educational Application for Mental Health Self-Monitoring After Bariatric Surgery: Development and Content Validation Study

**DOI:** 10.2196/92810

**Published:** 2026-08-12

**Authors:** Amanda Polaro, Roberta M Machado

**Affiliations:** 1 Hospital Israelita Albert Einstein São Paulo, São Paulo Brazil

**Keywords:** bariatric surgery, web-based applications, mental health, cognitive behavioral therapy, weight loss, mental disorders

## Abstract

**Background:**

Despite the effectiveness of bariatric surgery in the treatment of severe obesity, a substantial proportion of patients experience insufficient weight loss or weight regain over time. Evidence indicates that behavioral factors and mental health conditions play a central role in these outcomes, representing strategic targets for educational and technology-based self-monitoring interventions.

**Objective:**

This study aimed to develop and validate the content of a web-based application designed to support mental health self-monitoring and behavioral change among patients undergoing bariatric surgery.

**Methods:**

This is a formative research study focused on the development and content validation of a digital health educational intervention conducted in accordance with the systematic instructional design model, encompassing the phases of analysis, design/development, and validation. Content validation was performed by a panel of 13 experts using the Pasquali criteria. Interrater agreement was quantitatively assessed using the content validity index (CVI), considering the domains of clarity and relevance, with a minimum acceptable agreement threshold of ≥0.80.

**Results:**

The application was developed with 11 screens; it integrates validated psychometric instruments for the self-monitoring of key mental health conditions, including the Patient Health Questionnaire-9, the Generalized Anxiety Disorder-7, the modified Yale Food Addiction Scale 2.0, and the Alcohol Use Disorders Identification Test. In addition, the platform includes body weight monitoring, physical activity tracking, and access to educational content on healthy eating and mental health. The application incorporates motivational strategies such as goal setting, automated alerts, and encouragement of multidisciplinary follow-up. All screens demonstrated content validity in terms of relevance (item-level CVI [I-CVI]=1.00; scale-level CVI=1.00). For the clarity domain, only 1 screen presented an index below the predefined cutoff in the initial evaluation. After revision and adjustments, this screen was re-evaluated and achieved satisfactory agreement (I-CVI=0.92).

**Conclusions:**

The results indicate that the developed application demonstrates adequate content validity as a digital tool to support mental health care in the postoperative period of patients undergoing bariatric surgery. Although this study was limited to content validation, the findings support the formative development of a web-based platform for mental health self-monitoring and behavioral support in this population. Future studies should evaluate usability, acceptability, and clinical effectiveness in real-world settings.

## Introduction

Mental health problems are relatively common among adults undergoing bariatric surgery [1]. Although most studies indicate that the procedure is associated with short-term improvements in mental health disorders and symptoms, particularly depression and anxiety [2], these effects tend to be transient, with unfavorable outcomes emerging after weight stabilization [3]. Robust evidence demonstrates that changes in mental health status and maladaptive eating behaviors significantly influence postoperative weight regain [4,5].

In this context, intensive mental health monitoring should be considered an integral component of routine follow-up care for patients undergoing bariatric surgery [6,7], given the importance of early detection of adverse psychosocial outcomes [1]. Preventive approaches based on early identification of psychological and eating behavior changes represent sensitive strategies to mitigate unfavorable postoperative outcomes [4]. Moreover, continuous monitoring of mental health conditions combined with body weight surveillance [8] is an important factor in long-term weight maintenance [1].

With advances in digital health technologies, the integration of digital health tools has emerged as a promising strategy for health promotion and sustained patient engagement in healthy behaviors [9]. Digital health applications show substantial potential, particularly through self-monitoring features that enhance patient engagement and adherence to treatment [10]. However, studies specifically evaluating applications for mental health management and weight regain prevention in the postoperative period after bariatric surgery remain scarce.

A systematic review published in 2020 analyzing mobile apps designed for the management of overweight or obesity and depression or anxiety did not identify any app specifically developed for this population. The authors highlighted that interventions grounded in cognitive behavioral therapy principles appear to be the most appropriate for promoting sustainable long-term behavioral change [11].

Therefore, the development of digital tools that integrate health education, self-monitoring, and behavior change strategies tailored to the needs of patients undergoing bariatric surgery is highly relevant. Accordingly, this study aimed to develop and validate the content of a web-based educational application designed for the self-monitoring of mental health and body weight in patients undergoing bariatric surgery through expert panel evaluation. Such tools may contribute to strengthening self-care and self-regulation, as well as enabling the early identification of warning signs that indicate the need for specialized support.

## Methods

### Study Design

This methodological study involved the development and content validation of a digital health educational application guided by the principles of systematic instructional design, encompassing the phases of analysis, design, development, and validation. The content validation process followed the Pasquali framework and the recommendations of the COSMIN (Consensus-based Standards for the Selection of Health Measurement Instruments) guidelines for the evaluation of content validity [12].

### Ethical Considerations

This study was approved by the research ethics committee of Hospital Israelita Albert Einstein (7,318,672) in accordance with resolution number 466/12 of the Brazilian National Health Council. All participants involved in the validation phase received detailed information about the study objectives and procedures and provided written informed consent electronically prior to participation. Data were collected using the REDCap platform (Vanderbilt University), a secure electronic system widely used for data collection and management in research. To ensure participant privacy and confidentiality, all data were stored on the platform, with access restricted to the responsible researchers. No personally identifiable information was disclosed in any publications or presentations arising from this study. Participants did not receive financial compensation for their participation.

### Literature Review

A targeted review of the literature was conducted to identify scientific evidence on mental health conditions, eating behavior, weight regain, and self-monitoring strategies relevant to the postoperative bariatric population, providing the basis for the development of the application content. Searches were performed in the PubMed, LILACS, and SciELO databases on November 30, 2024, including studies published between 2007 and 2024. The search strategy combined controlled terms (MeSH) and free-text terms related to bariatric surgery, weight changes, mental health conditions, and mobile health technologies.

Study selection was conducted by 1 researcher in 2 stages: screening of titles and abstracts, followed by full-text review of potentially relevant studies. Studies were included if they addressed mental health, eating behavior, weight regain, behavioral factors, self-monitoring strategies, or digital health interventions relevant to the care of patients undergoing bariatric surgery. Articles unrelated to the study objectives, duplicate records, and publications without accessible full text were excluded. A total of 95 articles were identified, of which 11 were included to support the development of the intervention.

Given the limited availability of studies specifically addressing digital mental health support in bariatric populations, evidence from mobile health apps and digital behavioral interventions developed for broader health care settings was also considered when relevant to the objectives of the application.

In addition, complementary studies were identified through manual searches of reference lists to support the selection of validated self-administered instruments incorporated into the application and to inform the content validation process.

Finally, an exploratory analysis of existing digital apps focused on health education for patients undergoing bariatric surgery was conducted based on both the literature review and searches of commercially available applications in the Apple App Store and Google Play Store using keywords related to bariatric surgery, weight management, and mental health. The analysis emphasized functionalities related to weight regain prevention and mental health support. This step enabled the identification of gaps in currently available digital solutions and informed the definition of the functionalities proposed for the developed application.

### Application Design and Development

A total of 11 screens, corresponding to all functional modules of the application, were included in the content validation process. The application screens were developed based on the mental health conditions most frequently associated with the postoperative period following bariatric surgery, as well as the importance of continuous self-monitoring of body weight, physical activity, and mental health indicators. The organization of content and functionalities was planned considering the user’s journey within the application, aiming to provide intuitive, logical, and easy-to-understand navigation.

Initial wireframes were developed collaboratively with the contracted information technology team during meetings focused on defining screen layout, navigation flow, and content organization. Educational messages and supportive content were drafted by the research team based on the scientific literature and clinical experience in the follow-up of patients undergoing bariatric surgery.

### Platform Security, Privacy, and Identity

The application was developed as a web-based platform, accessible via a web address and hosted on a dedicated server with secure storage infrastructure. Access is granted through a username and password defined by the user, without requiring the entry of personally identifiable information, such as name, email, or official identification documents, thereby ensuring anonymity in platform use.

User-entered data are stored individually and linked exclusively to login credentials, with no access granted to other users, researchers, or health care professionals. No data are shared with third parties, and the system does not include a clinical monitoring dashboard or institutional access to user data. Communication between the user and the system is secured via HTTPS, ensuring protection of data in transit. Data are retained for as long as the user maintains an active account and may be deleted upon user request. Password recovery is performed through user-defined security questions. Access management is therefore the sole responsibility of the user, and maintaining the confidentiality of login credentials is essential.

The application was designed to ensure that server logs, IP addresses, device identifiers, and analytical use data are not collected from future users. This approach reflects the educational and supportive purpose of the application and respects ethical principles related to user autonomy and privacy.

At this stage, the technology was named Mcare, in which the letter M represents *mental health* and *monitoring*, while the term *care* emphasizes the human-centered, educational, and preventive nature of the intervention. Mcare was developed in Brazilian Portuguese.

### Mental Health Self-Monitoring

To enable the self-monitoring of key mental health conditions following bariatric surgery, 4 psychometric instruments with established international validity and validated Brazilian Portuguese versions were integrated into Mcare: the Patient Health Questionnaire-9 (PHQ-9) [13], Generalized Anxiety Disorder-7 (GAD-7) [14], Alcohol Use Disorders Identification Test (AUDIT) [15], and modified Yale Food Addiction Scale 2.0 (mYFAS 2.0) [16]. Details of the psychometric instruments and their respective purposes are presented in Multimedia Appendix 1 [13-16].

These instruments are widely used for mental health screening and monitoring and do not replace formal clinical diagnostic assessment. Mcare is a self-managed tool designed to support mental health self-monitoring and user awareness and does not provide active monitoring by health care professionals or replace routine clinical follow-up.

On the basis of score ranges established in the literature for each instrument, the application generates automated feedback and alerts whenever scores reach predefined cutoff thresholds. These messages are intended to provide educational guidance, encourage self-care practices, and suggest seeking professional support when appropriate. The original Portuguese feedback messages and their corresponding English translations are provided in Multimedia Appendix 2.

The application does not include mechanisms for automatic referral, third-party notification, or real-time monitoring, and users remain responsible for deciding whether to seek health care services.

Given the clinical relevance of item 9 of the PHQ-9, which assesses suicidal ideation, specific logic for this item has not yet been implemented in the current minimum viable product. However, this feature is planned for the next iteration of the application, prior to user testing. In cases of a positive response, the system will provide a differentiated alert, including immediate guidance to seek professional support and access to support resources, such as the Centro de Valorização da Vida (telephone helpline 188).

### Behavioral Monitoring

Mcare includes specific features for behavioral monitoring, such as periodic body weight recording with graphical visualization of weight trends over time. This feature aims to promote continuous body awareness and assist in early detection of significant weight fluctuations that may indicate risk of weight regain.

In addition, users can log their weekly physical activity duration, set goals, and specify the type of exercise performed, with their progress compared against World Health Organization recommendations [17]. The system provides motivational messages and positive reinforcement to encourage adherence to an active lifestyle.

The application also provides educational content on dietary quality based on clinical guidelines for patients undergoing bariatric surgery, including practical guidance on balanced meal composition [18]. As a complementary strategy, audiovisual resources focused on emotional regulation, anxiety management, and psychological coping strategies were incorporated, offering immediate digital support during moments of emotional vulnerability.

### Main Interfaces of Mcare

**Figure 1:**
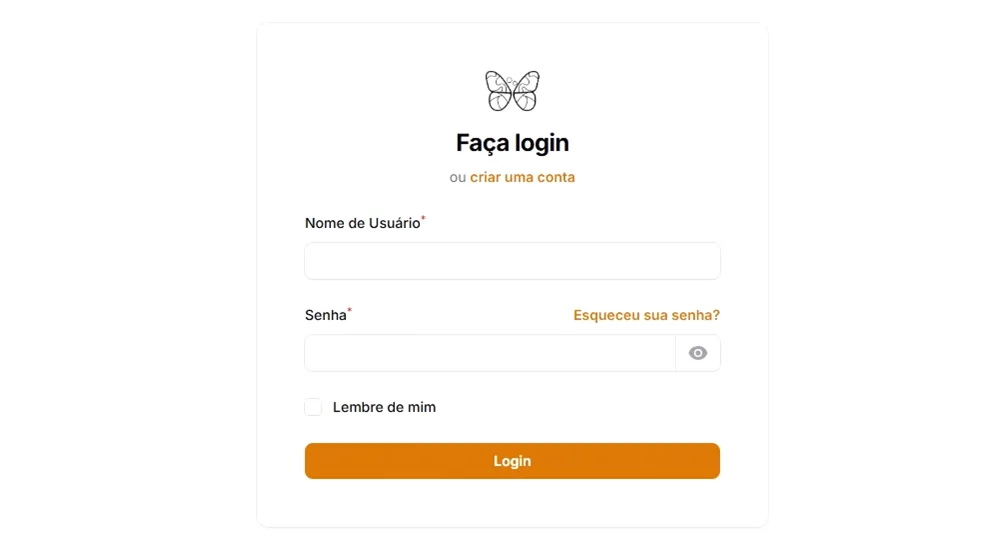
Login interface of the Mcare web-based application. This interface allows users to create login credentials and access the application without requiring identifiable personal information or email registration.

**Figure 2:**
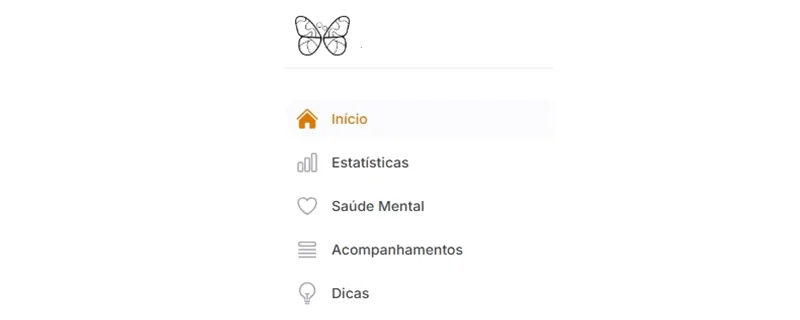
Initial interface of the Mcare web-based application showing the modules available to users.

**Figure 3:**
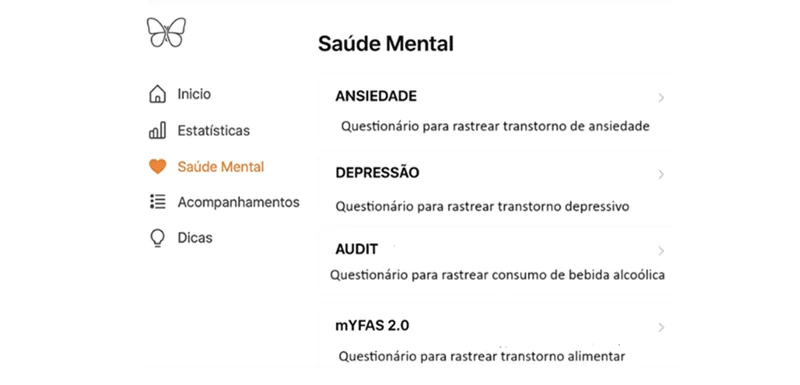
Mental health self-monitoring interface of the Mcare web-based application using validated psychometric screening instruments. This interface enables self-monitoring of mental health conditions after bariatric surgery through validated self-administered questionnaires for the early identification of depressive symptoms, anxiety, food addiction, and alcohol misuse.

**Figure 4:**
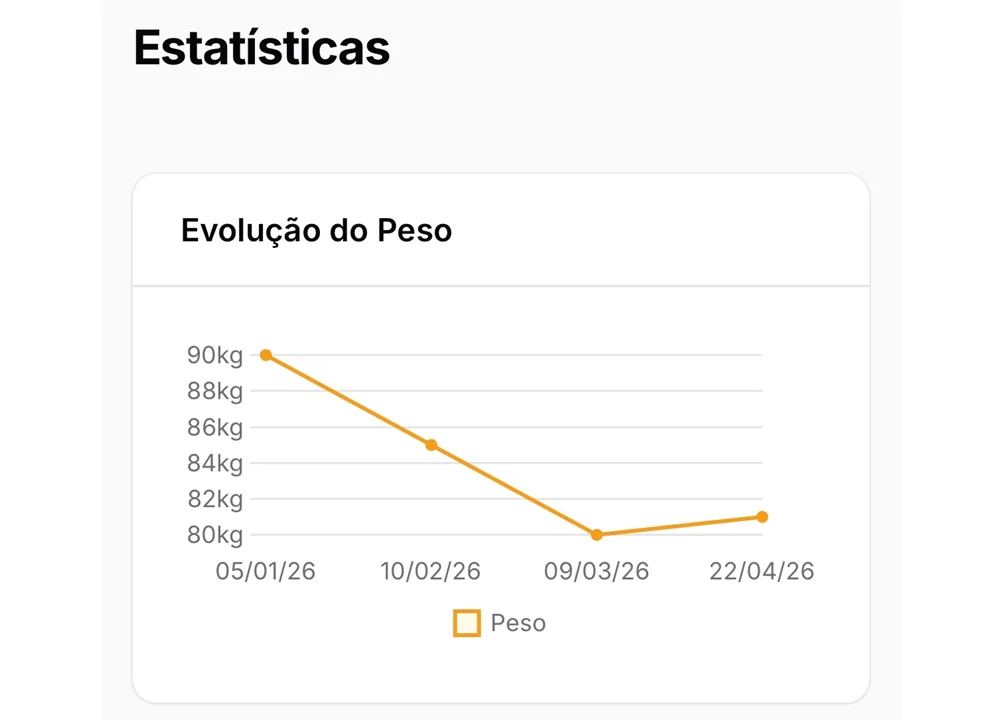
Body weight monitoring interface of the Mcare web-based application, enabling longitudinal tracking of self-reported weight data. Data entered into Mcare are self-reported by the user. Body weight can be recorded at different time points over time. The graphical representations presented in Mcare are illustrative and intended to demonstrate longitudinal monitoring. They do not necessarily reflect ideal clinical patterns or constitute treatment recommendations.

**Figure 5:**
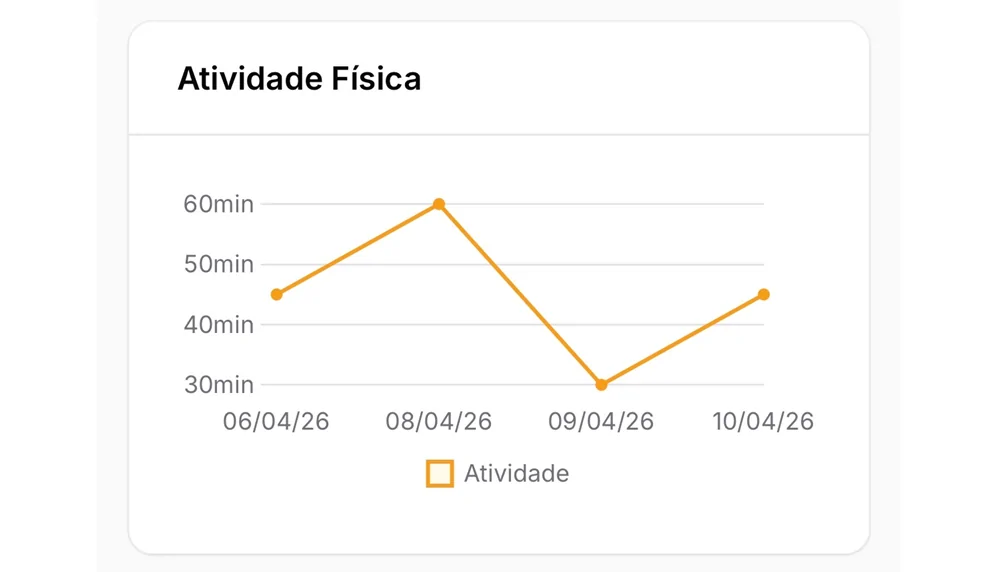
Behavioral monitoring module interface of the Mcare web-based application for logging physical activity duration. Physical activity duration is recorded based on the activity performed and may include individual sessions or cumulative activity within the same day.

Figures 1-5 present the main interfaces of Mcare, corresponding to its functional modules. The images are illustrative and depict the minimum viable product.

As shown in Figure 2, Mcare was structured into 5 main modules: home, statistics, mental health, tracking, and tips. The home module presents the main interface of the application, allowing navigation across available functionalities. The statistics module enables graphical visualization of body weight trends and physical activity duration, supporting longitudinal user monitoring. The mental health module integrates validated psychometric instruments (PHQ-9, GAD-7, AUDIT, and mYFAS 2.0), enabling the self-monitoring of mental health–related symptoms. The tracking module is intended for user input of personal data, including body weight (with date) and weekly physical activity duration. The tips module provides educational content aimed at health promotion, including guidance on nutrition and emotional regulation techniques such as meditation and mindful breathing.

### Validation Process

Content validation was conducted through expert evaluation based on the criteria proposed by Pasquali (refer to the study by Medeiros et al [19]) and the COSMIN recommendations for content validity [12], using an iterative process to refine the application screens. The evaluation panel comprised 3 distinct groups: health care professionals involved in the clinical follow-up of patients in the postoperative period of bariatric surgery; patients who had undergone bariatric surgery within the past 5 years, contributing the end-user perspective; and information technology specialists with experience in application development.

Health and technology experts were recruited by convenience through searches of scientific publications and curriculum analysis on the Lattes Platform. Eligibility criteria were adapted from the Fehring model [20], considering academic qualifications, scientific production, teaching experience, participation in research projects, and practical experience in the field of interest, with a minimum score of 5 points required for inclusion in the expert panel. Patient evaluators were recruited from users previously registered in the Barilife application, developed by the Brazilian Society of Bariatric and Metabolic Surgery. Eligible participants included men and women aged ≥18 years who were within 5 years after bariatric surgery.

After agreeing to participate and providing written informed consent, the evaluators received the application prototype and the evaluation instrument via email through the REDCap platform. The questionnaire assessed each screen in terms of clarity and relevance using a 4-point Likert scale [21] (Table 1), with scores of 0 and 1 classified as “not adequate” and scores of 2 and 3 as “adequate,” in addition to including open-ended fields for suggestions and comments. Participants were given 15 days to complete the evaluation. The first round of evaluation took place from September 29, 2025, to October 13, 2025.

**Table 1:** Four-point Likert scale used by experts to assess the clarity and relevance of the application screens during the content validation process.

Score	Clarity	Relevance
0	Not clear	Not relevant
1	Slightly clear	Slightly relevant
2	Clear	Relevant
3	Very clear	Very relevant

Screens that did not meet the predefined content validity criteria were revised based on reviewer feedback and submitted for a second round of evaluation, conducted between November 3, 2025, and November 18, 2025. All reviewers analyzed all application screens. Responses were weighted equally across participant groups (health care professionals, information technology specialists, and patients) without differential weighting between groups. Equal weighting was intentionally adopted to standardize the evaluation process and facilitate comparisons between domains. This approach ensured consistency in data interpretation, allowing each group to contribute according to their specific expertise.

### Statistical Analysis

Content validity was assessed using the content validity index (CVI), including the item-level CVI (I-CVI) and the scale-level CVI (S-CVI). For each item, the I-CVI was calculated as the proportion of evaluators rating the item as adequate (scores 2 or 3) relative to the total number of evaluators. The S-CVI was calculated as the average of the I-CVI values across all evaluated screens (S-CVI or average). Values ≥0.80 were considered indicative of satisfactory content validity [22].

Mean clarity and relevance scores were also calculated for each application screen based on the 4-point Likert scale responses.

## Results

### Sample Characteristics

A total of 18 individuals were invited to participate in the expert review panel according to the study inclusion criteria, of whom 13 completed the evaluation instruments. The sample comprised 3 distinct groups: 4 health care professionals (1 nutritionist, 1 psychologist, 1 endocrinologist, and 1 bariatric surgeon), 4 information technology professionals with experience in application development, and 5 patients who had undergone bariatric surgery.

### Health Care Professionals’ Profile

Among the 4 health care professionals, 2 (50%) held a master’s degree, 1 (25%) had served as a thesis adviser, and collectively they had published 11 scientific articles related to the study topic. All health care professionals were involved in research projects or research groups in the health field, and 3 (75%) health care professionals reported experience in higher education teaching. All evaluators in this group reported clinical practice in the postoperative follow-up of patients undergoing bariatric surgery, with a mean of 23.25 (SD 21.93) years of professional experience.

### Information Technology Specialists’ Profile

Among the 4 information technology specialists, 1 (25%) held a doctoral degree and 1 (25%) had served as a thesis adviser. All information technology specialists reported prior experience in the development of health-related applications, with a mean of 13.75 years of professional experience.

### Characteristics of Patient Evaluators

The patient evaluator group ranged in age from 26 to 53 years. Of the 5 participants, 4 (80%) were female. Time since bariatric surgery ranged from 1 year and 2 months to 2 years and 9 months. Notably, 2 patients (40%) reported not having received psychological follow-up during the postoperative period.

### Evaluation of Mcare Screens

A total of 11 application screens were analyzed. In the first round of evaluation, all screens demonstrated maximum agreement for relevance (I-CVI=1.00), with a global S-CVI of 1.00, indicating consensus among evaluators regarding the clinical and scientific adequacy of the content.

Regarding clarity, most screens received satisfactory scores in the initial assessment (I-CVI ranging from 0.92 to 1.00), with an overall S-CVI of 0.94. Only screen 6 presented an index below the established limit (I-CVI=0.77). Analysis by evaluator group showed that all patients rated the clarity of all screens as adequate. Among health care professionals, only screen 6 received 2 ratings, indicating inadequate clarity mainly due to the use of unexplained acronyms. In the technology expert group, 5 screens received 1 inadequate rating each. Qualitative feedback from this group highlighted opportunities to improve visual organization, including adjustments to color schemes, the addition of explanatory text for graphics, supplementary text accompanying each tip, and clarification of the acronyms used on screen 6. On the basis of this feedback, the screen was revised to include complete explanations of terms and improve overall clarity.

In the first round of evaluation, the overall mean clarity score was 2.60 (SD 0.66), while the overall mean relevance score was 2.77 (SD 0.42). Across the evaluated screens, mean clarity scores ranged from 2.15 to 2.77, whereas mean relevance scores ranged from 2.58 to 2.84.

### Second Round of Evaluation

Screen 6 was revised to include complete descriptions of the acronyms and improve the intelligibility of the interface. After the revision, this screen underwent a second round of evaluation, conducted from November 3, 2025, to November 18, 2025, and obtained satisfactory agreement regarding clarity (I-CVI=0.92) and relevance (I-CVI=1.00), meeting the criteria for final validation. After the second round, the S-CVI for clarity reached 0.96. Agreement across screens is presented in Table 2.

**Table 2:** Judges’ agreement regarding clarity and relevance of the application screens.

Screen and evaluated dimension			I-CVI^a^—first round		Modification required		Final I-CVI
**1**							
	Relevance	1.00		No		1.00	
	Clarity	1.00		No		1.00	
**2**							
	Relevance	1.00		No		1.00	
	Clarity	0.92		No		0.92	
**3**							
	Relevance	1.00		No		1.00	
	Clarity	1.00		No		1.00	
**4**							
	Relevance	1.00		No		1.00	
	Clarity	1.00		No		1.00	
**5**							
	Relevance	1.00		No		1.00	
	Clarity	1.00		No		1.00	
**6**							
	Relevance	1.00		No		1.00	
	Clarity	0.77		Yes		0.92	
**7**							
	Relevance	1.00		No		1.00	
	Clarity	0.92		No		0.92	
**8**							
	Relevance	1.00		No		1.00	
	Clarity	0.92		No		0.92	
**9**							
	Relevance	1.00		No		1.00	
	Clarity	0.92		No		0.92	
**10**							
	Relevance	1.00		No		1.00	
	Clarity	0.92		No		0.92	
**11**							
	Relevance	1.00		No		1.00	
	Clarity	1.00		No		1.00	

^a^I-CVI: item-level content validity index.

Following revision of screen 6, the overall mean clarity score was 2.63 (SD 0.66), while the overall mean relevance score was 2.77 (SD 0.42). Across the evaluated screens, mean clarity score improved from 2.46 to 2.77. Mean clarity and relevance scores for all application screens are presented in Table 3.

**Table 3:** Mean clarity and relevance scores of the application screens.

Screen	Evaluation round	Clarity score, mean (SD)	Relevance score, mean (SD)
1	Round 1	2.54 (0.52)	2.61 (0.51)
2	Round 1	2.69 (0.63)	2.69 (0.48)
3	Round 1	2.54 (0.52)	2.58 (0.51)
4	Round 1	2.46 (0.44)	2.84 (0.38)
5	Round 1	2.77 (0.48)	2.84 (0.48)
6	Round 1	2.15 (0.86)	2.77 (0.44)
6	Round 2	2.46 (0.88)	2.77 (0.48)
7	Round 1	2.69 (0.75)	2.77 (0.48)
8	Round 1	2.61 (0.85)	2.77 (0.38)
9	Round 1	2.61 (0.85)	2.77 (0.48)
10	Round 1	2.61 (0.85)	2.84 (0.39)
11	Round 1	2.69 (0.48)	2.84 (0.38)

## Discussion

### Key Findings

This study achieved its objective of developing and validating the content of an educational application designed to support the self-monitoring of mental health and body weight in patients undergoing bariatric surgery. The Mcare application was developed based on scientific evidence; integrated validated psychometric instruments for screening mental health conditions prevalent in this population; and demonstrated adequate content validity following multidisciplinary evaluation by health care professionals, information technology specialists, and patients who had undergone bariatric surgery. The results suggested that the content was considered relevant and that the application screens achieved satisfactory levels of clarity after refinement.

### Comparison With the Literature and Clinical Implications

The satisfactory content validity demonstrated by Mcare reinforces the relevance of integrating mental health self-monitoring into long-term postoperative bariatric care. Previous studies have reported persistent or recurrent symptoms of depression and anxiety, loss of control eating, emotional eating, substance misuse, and alcohol-related problems after surgery [2,3]. These conditions have been associated with suboptimal weight loss trajectories, weight regain, lower treatment adherence, and poorer quality of life [5]. Collectively, these findings highlight the importance of continuous psychological follow-up throughout long-term postoperative care [23].

However, maintaining long-term follow-up remains challenging in clinical practice. In this context, digital health technologies have emerged as promising strategies to enhance patient monitoring and support. Previous evidence has shown that smartphone-based self-monitoring is a feasible and acceptable approach among individuals with psychiatric disorders [24]. In addition to increasing awareness of symptoms, this strategy may facilitate the early identification of symptom worsening and promote mental health self-management [24]. Although the literature has focused predominantly on interventions developed for mobile devices, it is reasonable to hypothesize that the potential benefits of digital self-monitoring may also extend to web-based applications, as both share core functionalities and may serve similar roles in symptom monitoring.

A systematic review of apps targeting the comanagement of obesity or overweight and depression or anxiety identified a limited number of studies and found that most available tools focused primarily on weight and physical activity monitoring, with limited attention to psychological aspects [11]. These findings underscore the need for digital solutions that integrate the monitoring of mental health symptoms and related behaviors, as proposed in the present study.

To address this unmet need, the application developed in this study integrates mental health and behavioral self-monitoring within a single online platform. The selection of the PHQ-9, GAD-7, AUDIT, and mYFAS 2.0 instruments was based on their widespread use in clinical and research settings, their well-established psychometric properties, and their feasibility for administration in digital environments. These instruments assess some of the most clinically relevant psychological conditions associated with postoperative outcomes, including depressive symptoms, anxiety, dysfunctional eating behaviors, and problematic alcohol use [2]. Importantly, these instruments are screening tools and are not intended to establish clinical diagnoses. Their incorporation into a self-administered digital platform may facilitate routine symptom monitoring and support the early identification of changes, potentially promoting self-care and mental health self-management.

Despite these potential advantages, an important limitation of the current version of the application is the absence of a specific protocol for managing positive responses to item 9 of the PHQ-9, which assesses suicidal ideation. Reliance solely on the total PHQ-9 score may be insufficient to identify individuals at risk in unsupervised settings. Therefore, future versions should incorporate automated safety procedures, including immediate risk alerts, crisis support information, and guidance for seeking professional mental health care. For users in Brazil, this could include referral information for the Centro de Valorização da Vida, a national suicide prevention and emotional support service available through telephone helpline 188. Such modifications are essential before implementing the application in real-world settings.

Another relevant feature of the proposed application is the inclusion of self-monitoring components targeting behavioral factors amenable to change. Previous research has identified poor adherence to dietary recommendations, including excessive alcohol consumption, maladaptive eating behaviors, lack of ongoing follow-up with the bariatric care team, and insufficient physical activity as major factors associated with weight regain [25]. In addition, individuals who engage in long-term self-weighing behaviors tend to achieve better clinical outcomes [26].

Accordingly, the inclusion of features such as graphical visualization of weight trends, physical activity tracking and encouragement, educational content on diet quality, and audiovisual resources aimed at emotional regulation may strengthen self-regulation and self-care strategies during long-term follow-up, thereby supporting the maintenance of healthy behaviors.

Although the current version demonstrated satisfactory content validity, future studies should include accessibility and usability testing among users with different levels of literacy and health literacy. Previous research has shown that limited health literacy may negatively affect the ability to access, understand, and use health information, potentially reducing engagement with digital health interventions [27]. Therefore, ensuring that Mcare is understandable and usable across different literacy levels will be important to maximize its reach and effectiveness.

### Methodological Strengths

The validation process included 13 judges with diverse backgrounds, enabling a multidisciplinary and user-centered evaluation. The simultaneous participation of health care professionals, information technology specialists, and patients who had undergone bariatric surgery strengthened the clinical, functional, and experiential adequacy of the digital intervention. Furthermore, the use of the Pasquali criteria [19], COSMIN recommendations [12], and the CVI reinforces the methodological rigor of the study. Considering recommendations regarding the minimum number of judges (ranging from 6 to 20 participants with appropriate representation across groups) [20], the validation process can be considered methodologically appropriate for initial content validation and provided evidence supporting the relevance, clarity, and adequacy of the proposed content.

### Limitations

This study has some limitations. First, the validation focused on the application’s content and did not assess clinical outcomes, long-term usability, or the effectiveness of the intervention. In addition, the current version of the application does not include a specific safety protocol for positive responses to item 9 of the PHQ-9, which assesses suicidal ideation. Although users receive automated feedback based on overall questionnaire scores, future versions should incorporate dedicated risk alerts and referral pathways to enhance patient safety in unsupervised settings. Furthermore, the sample size of evaluators, although appropriate for content validation studies, limits the generalizability of the findings. Future studies should investigate user engagement, user experience, and the impact of the application on clinical and psychological outcomes, as well as on weight maintenance after bariatric surgery.

An additional implementation consideration relates to the password recovery mechanism, which is currently based on security questions and may raise usability and security concerns. Furthermore, patient evaluators were recruited from the Barilife platform, which may have introduced selection bias toward individuals who are more digitally engaged and familiar with online health resources.

### Conclusions

This study demonstrated the content validity of a web-based application designed for mental health self-monitoring among patients undergoing bariatric surgery. The content was considered clear, relevant, and appropriate by expert judges and patient evaluators, supporting the formative development of Mcare. After implementing the necessary refinements, including safety-related adjustments, future studies involving end users will be required to evaluate usability, acceptability, and practical applicability before investigating the application’s clinical effectiveness.

## Appendix

Multimedia Appendix 1 Psychometric instruments integrated into the application for self-monitoring of mental health in patients undergoing bariatric surgery.

Multimedia Appendix 2 Original Portuguese feedback messages and corresponding English translations used in the Mcare application.

## Abbreviations

AUDIT: Alcohol Use Disorders Identification Test
COSMIN: Consensus-based Standards for the Selection of Health Measurement Instruments
CVI: content validity index
GAD-7: Generalized Anxiety Disorder-7
I-CVI: item-level content validity index
mYFAS 2.0: modified Yale Food Addiction Scale 2.0
PHQ-9: Patient Health Questionnaire-9
S-CVI: scale-level content validity index

## Data Availability

The deidentified validation data that support the findings of this study, including the individual ratings used to calculate the content validity index, are not publicly available due to confidentiality and privacy considerations. However, they may be made available by the corresponding author upon reasonable request, subject to approval by the research ethics committee of Hospital Israelita Albert Einstein.
