# Supplementary material for: Web-Based Educational Application for Mental Health Self-Monitoring After Bariatric Surgery: Development and Content Validation Study
Source: JMIR Form Res. 2026 Aug 12;10:e92810. doi: 10.2196/92810 (PMC13519511; doi:10.2196/92810)
Supplement: Supplementary file 1 [file formative_v10i1e92810_app1.docx]

**Multimedia Appendix 1.** Psychometric instruments integrated into the application for self-monitoring of mental health in patients undergoing bariatric surgery

| Patient Health Questionnaire-9 (PHQ-9) [13] | Screening of depressive symptoms | 9 | Score <10 (minimal or mild symptoms): “Congratulations on investing in self-care. Paying attention to your mental health is always a valuable choice.”  Score ≥10 (moderate to severe symptoms): “Seek help from a specialized professional to support your mental health care. You are not alone! Asking for help is an act of courage.” |
| --- | --- | --- | --- |
| Generalized Anxiety Disorder-7 (GAD-7) [14] | Generalized anxiety screening | 7 | Score <10 (minimal or mild symptoms): “Congratulations on investing in self-care. Paying attention to your mental health is always a valuable choice.”  Score ≥10 (moderate to severe symptoms): “Anxiety may be affecting your daily life. You are not alone! Seeking support is an important step.” |
| Alcohol Use Disorders Identification Test (AUDIT) [15] | Identification of harmful alcohol use patterns | 10 | Score <8 (low risk): “Congratulations on investing in self-care. Paying attention to your mental health is always a valuable choice.”  Score ≥8 (risky use): “Your alcohol consumption pattern suggests potential health risk. Small changes can make a difference. Consider reducing frequency and quantity.”  Score ≥16 (harmful use or probable dependence): “Your alcohol use may be negatively affecting your health. You are not alone! Seeking professional support can be an important step.” |
| Modified Yale Food Addiction Scale 2.0 (mYFAS 2.0) [16] | Screening for signs similar to food addiction | 13 | No diagnosis: does not meet criteria (0-1 symptoms): “Your results do not indicate clinically significant signs similar to those observed in food addiction. Maintaining this awareness is a valuable step toward long-term well-being.”  Mild: meets diagnostic criteria (2-3 symptoms): ”Your results suggest mild signs similar to those observed in food addiction. Paying attention to these patterns and adopting healthy coping strategies may help prevent progression.”  Moderate: meets diagnostic criteria (4–5 symptoms): ”Your results suggest a moderate pattern of addictive-like eating behaviors. Monitoring your eating behaviors and seeking guidance from a qualified health professional is recommended.”  Severe: meets diagnostic criteria (≥6 symptoms): ”Your results indicate a severe pattern of addictive-like eating behaviors. You are not alone—seeking support from a specialized health professional is strongly recommended for your well-being.” |

**References**

13. Santos IS, Tavares BF, Munhoz TN, Almeida LSP, Silva NTB, Tams BD, et al. Sensitivity and specificity of the Patient Health Questionnaire-9 (PHQ-9) among adults from the general population. Cad Saude Publica. 2013;29(8):1533-1543. doi:10.1590/0102-311X00144612

14. Moreno AL, DeSousa DA, Souza AMFLP, Manfro GG, Salum GA, Koller SH, et al. Factor structure, reliability, and item parameters of the GAD-7 among Brazilian adults. Trends Psychiatry Psychother. 2016;38(2):100-107. [doi:10.9788/TP2016.1-25](https://doi.org/10.9788/TP2016.1-25)

15. Moretti-Pires RO, Corradi-Webster CM. Adaptação e validação do Alcohol Use Disorder Identification Test (AUDIT) para população ribeirinha do interior da Amazônia, Brasil. Cad Saude Publica. 2011;27(3):497-509. doi:10.1590/S0102-311x2011000300010

16. Schulte EM, Gearhardt AN. Development of the modified Yale Food Addiction Scale version 2.0. Eur Eat Disord Rev. 2017;25(4):302-308. doi:10.1002/erv.2515
