# Supplementary material for: Web-Based Educational Application for Mental Health Self-Monitoring After Bariatric Surgery: Development and Content Validation Study
Source: JMIR Form Res. 2026 Aug 12;10:e92810. doi: 10.2196/92810 (PMC13519511; doi:10.2196/92810)
Supplement: Supplementary file 2 [file formative_v10i1e92810_app2.docx]

**Multimedia Appendix 2.** Original Portuguese feedback messages and corresponding English translations used in the Mcare application

| **Instrument and score category** | **Original text in Portuguese** | **English translation** |
| --- | --- | --- |
| PHQ-9  (score <10) | "Parabéns por investir no autocuidado. Cuidar da sua saúde mental é sempre uma escolha valiosa." | "Congratulations on investing in self-care. Paying attention to your mental health is always a valuable choice." |
| PHQ-9  (score ≥10) | "Procure ajuda de um profissional especializado para apoiar o cuidado da sua saúde mental. Você não está sozinho! Pedir ajuda é um ato de coragem." | "Seek help from a specialized professional to support your mental health care. You are not alone! Asking for help is an act of courage." |
| GAD-7  (score <10) | "Parabéns por investir no autocuidado. Cuidar da sua saúde mental é sempre uma escolha valiosa." | "Congratulations on investing in self-care. Paying attention to your mental health is always a valuable choice." |
| GAD-7  (score ≥10) | "A ansiedade pode estar afetando sua vida diária. Você não está sozinho! Buscar apoio é um passo importante." | "Anxiety may be affecting your daily life. You are not alone! Seeking support is an important step." |
| AUDIT  (score <8) | "Parabéns por investir no autocuidado. Cuidar da sua saúde mental é sempre uma escolha valiosa." | "Congratulations on investing in self-care. Paying attention to your mental health is always a valuable choice." |
| AUDIT  (score ≥8) | "Seu padrão de consumo de álcool sugere risco potencial à saúde. Pequenas mudanças podem fazer diferença. Considere reduzir a frequência e a quantidade." | "Your alcohol consumption pattern suggests potential health risk. Small changes can make a difference. Consider reducing frequency and quantity." |
| AUDIT  (score ≥16) | "O uso de álcool pode estar afetando negativamente sua saúde. Você não está sozinho! Buscar apoio profissional pode ser um passo importante." | "Your alcohol use may be negatively affecting your health. You are not alone! Seeking professional support can be an important step." |
| mYFAS 2.0  (0–1 symptoms) | "Seus resultados não indicam sinais clinicamente significativos semelhantes aos observados na dependência alimentar. Manter essa atenção é um passo importante para o bem-estar em longo prazo." | "Your results do not indicate clinically significant signs similar to those observed in food addiction. Maintaining this awareness is a valuable step toward long-term well-being." |
| mYFAS 2.0  (2–3 symptoms) | "Seus resultados sugerem sinais leves semelhantes aos observados na dependência alimentar. Prestar atenção a esses padrões e adotar estratégias saudáveis pode ajudar a prevenir sua progressão." | "Your results suggest mild signs similar to those observed in food addiction. Paying attention to these patterns and adopting healthy coping strategies may help prevent progression." |
| mYFAS 2.0  (4–5 symptoms) | "Seus resultados sugerem um padrão moderado de comportamentos alimentares semelhantes ao vício. Recomenda-se monitorar os hábitos alimentares e buscar orientação de um profissional de saúde qualificado." | "Your results suggest a moderate pattern of addictive-like eating behaviors. Monitoring your eating behaviors and seeking guidance from a qualified health professional is recommended." |
| mYFAS 2.0  (≥6 symptoms) | "Seus resultados indicam um padrão grave de comportamentos alimentares semelhantes ao vício. Você não está sozinho — buscar apoio de um profissional de saúde especializado é fortemente recomendado para o seu bem-estar." | "Your results indicate a severe pattern of addictive-like eating behaviors. You are not alone—seeking support from a specialized health professional is strongly recommended for your well-being." |
